# Effect of extracorporeal hemoadsorption in critically ill patients with COVID-19: A narrative review

**DOI:** 10.3389/fimmu.2023.1074465

**Published:** 2023-02-03

**Authors:** Kaixi Chang, Yupei Li, Zheng Qin, Zhuyun Zhang, Liya Wang, Qinbo Yang, Jiwen Geng, Ningyue Deng, Shanshan Chen, Baihai Su

**Affiliations:** ^1^ Department of Nephrology, West China Hospital, Sichuan University, Chengdu, China; ^2^ Med-X Center for Materials, Sichuan University, Chengdu, China; ^3^ Med+ Biomaterial Institute of West China Hospital, Sichuan University, Chengdu, China

**Keywords:** COVID-19, hemoadsorption, cytokine storm, inflammatory mediators, immunomodulatory, blood purification

## Abstract

COVID-19 has been affecting the world unprecedentedly and will remain widely prevalent due to its elusive pathophysiological mechanism and the continuous emergence of new variants. Critically ill patients with COVID-19 are commonly associated with cytokine storm, multiple organ dysfunction, and high mortality. To date, growing evidence has shown that extracorporeal hemoadsorption can exert its adjuvant effect to standard of care by regulating immune homeostasis, reducing viremia, and decreasing endotoxin activity in critically ill COVID-19 cases. However, the selection of various hemofilters, timing of initiation and termination of hemoadsorption therapy, anticoagulation management of extracorporeal circuits, identification of target subgroups, and ultimate survival benefit remain controversial. The purpose of this narrative review is to comprehensively summarize the rationale for the use of hemoadsorption in critically ill patients with COVID-19 and to gather the latest clinical evidence in this field.

## 1 Introduction

The current pandemic of coronavirus disease 2019 (COVID-19) is caused by severe acute respiratory syndrome coronavirus 2 (SARS-CoV-2), with a spectrum of disease severity ranging from mild symptoms to critical illness. A recent systematic analysis found that the number of people who died from COVID-19 from Jan 1, 2020, to Dec 31, 2021 globally reached 18.2 million (1), which was much higher than the WHO official statistics (2).

The pathophysiological changes in the course of COVID-19 can be prevented and treated through various approaches. Efforts to develop effective means of prevention and treatment mainly target the host immune response to COVID-19 (3). For example, as the key to limiting SARS-CoV-2 transmission, real-world data have shown that vaccination has considerable effectiveness against severe disease and hospitalization despite the slow vaccination rate in some regions (4, 5). Antiviral medications such as remdesivir, and anti-inflammatory regimens such as corticosteroids, interleukin-6 (IL-6) inhibitors, and Janus kinase inhibitors have also been reported to improve clinical outcomes in hospitalized COVID-19 patients in specific subgroups (6–9). The emergence of new multiple variant infections indicates that protection against severe disease is really anticipated (10). Additionally, the reduction in the infection-fatality ratio in the postvaccination era should be further evaluated (11). Post-COVID syndrome, which is closely related to host immune dysfunction, has also attracted much attention recently (12).

Along with the characteristics and disease course of critically ill COVID-19 patients continuously evolving throughout the pandemic (13), we should note that treatment strategies still remain limited in a subgroup of critically ill COVID-19 patients with cytokine storm and multiple organ dysfunction syndrome (MODS), such as acute kidney injury (AKI) and acute respiratory distress syndrome (ARDS) (3). The truth is that the effectiveness of several explored therapeutic approaches, including antiplatelet agents and high-dose convalescent plasma, on the survival of critically ill COVID-19 patients is not promising (14–16). The conflicting study outcomes of anti-interleukin drugs also remind us that broader immunoregulation in severe patients is still required to prevent malignant disease progression (17).

Extracorporeal hemoadsorption, an important adjuvant treatment to standard of care, has been used in various critical care settings during the past two decades (18). Accumulating evidence collectively shows that the selective or nonselective removal of multiple inflammatory mediators and circulating toxins from the bloodstream during hemoadsorption sessions has an immediate effect on the regulation of host inflammatory response, but the evidence for beneficial effects is uncertain (19). More recently, new indications are developing in this field, and novel hemofilters are available for clinical use (20). An early systematic review recommended against the indiscriminate use of extracorporeal hemoadsorption in critically ill COVID-19 patients outside of investigational clinical trials. However, this analysis only included low-quality case series and observational studies with no randomized studies included (21). In contrast, another narrative review that included 16 studies (including a controlled trial) demonstrated that hemoadsorption therapy is an alternative salvage treatment method in critically ill COVID-19 patients, but it also has methodological shortcomings in data analysis and thus still needs to be supported by stronger evidence (22). Meanwhile, previously published narrative reviews were mostly based on previous practical experience with other diseases such as sepsis, severe acute respiratory syndrome and middle east respiratory syndrome (MERS) instead of COVID-19 (23, 24).

Herein, we summarize the rationale and the latest evidence for hemoadsorption that are exclusively applied in the specific context of COVID-19 until January 20, 2023. We also discuss perspectives for future research design and clinical application of hemoadsorption-based techniques in critically ill COVID-19 patients.

## 2 Rationale for the use of hemoadsorption in severe COVID-19 patients

**Figure 1:**
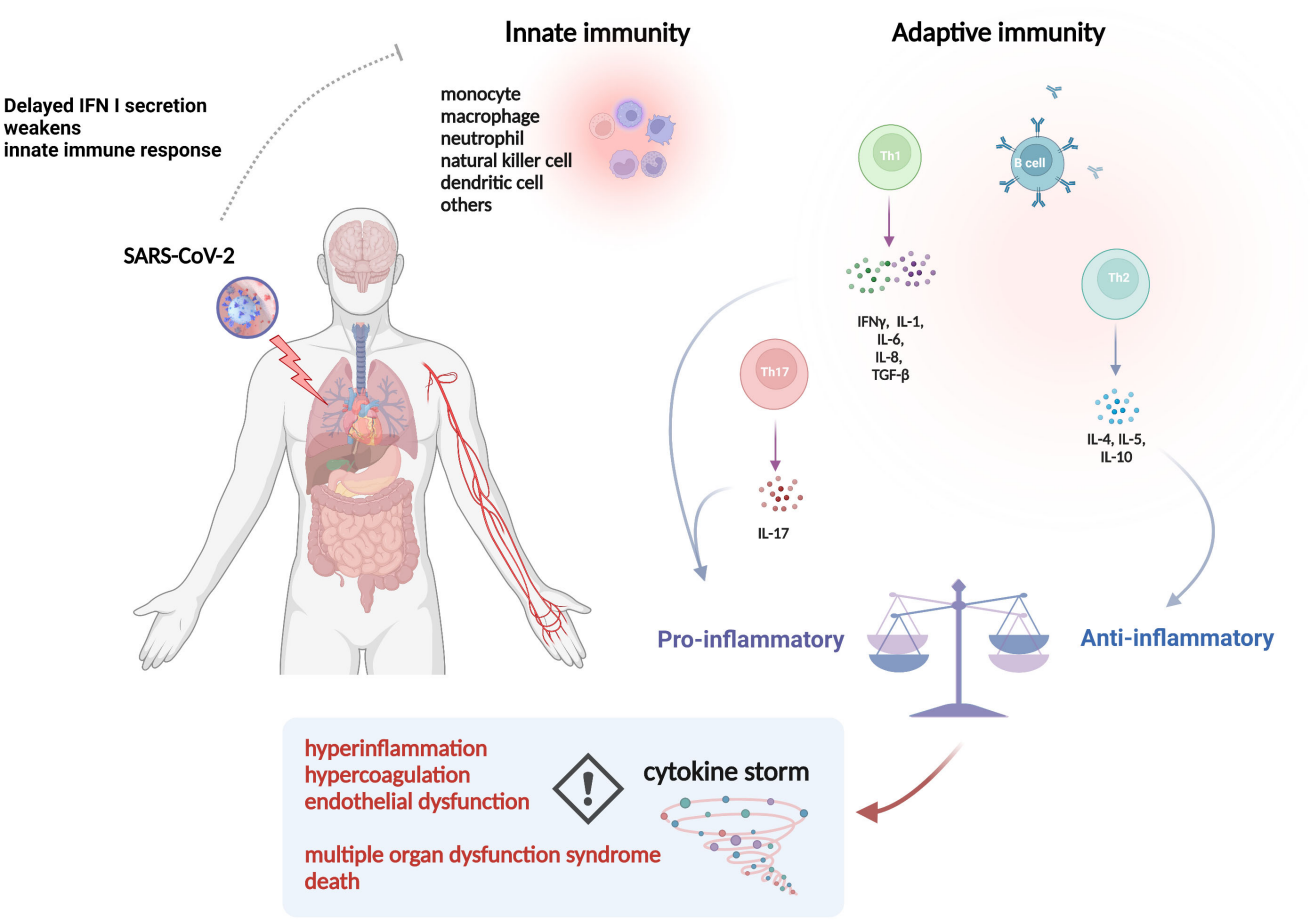
The pathophysiology of inflammatory events in COVID-19. Abbreviations: IFN, interferon; SARS-CoV-2, severe acute respiratory syndrome coronavirus 2; IL, interleukin; TGF, transforming growth factor. The picture was generated using BioRender software.

SARS-CoV-2 is a highly pathogenic virus. As shown in **Figure 1**, when SARS-CoV-2 invades the body, the first responder is innate immunity, like monocytes, macrophages, neutrophils, dendritic cells, natural killer cells, and then adaptive immunity, like T helper 1 (Th1), Th2 cells and B cells (25). Innate immune responses induced by pattern recognition receptors (PRRs) signaling activate effector cells to mediate viral clearance (26). Interferons (IFNs), classified as interferon I (IFN I), IFN II (IFN-γ) and IFN III, are critical in the initiation of the innate immune response, while delayed IFN I secretion induced by SARS-CoV-2 will reduce chemotaxis, leading to a weakened innate immune response (27). Typically, the adaptive immune response in COVID-19 patients shifts to the Th2 phenotype, and Th2 cells exerts anti-inflammatory effects by secreting cytokines such as IL-4, IL-5 and IL-10, which contributes to the control of SARS-CoV-2 infection rapidly (28). While the weakened innate immune response will in turn lead to enhanced viral replication and hyperactivation of Th1 cells, which subsequently activates macrophages by releasing IFN-γ, thus causing the production and secretion of IL-1, IL-6, IL-8 and transforming growth factor (TGF-β), the latter of which activate Th17 cells to secrete IL-17, and together generate a cytokine storm, which is characterized by an aberrant, rapid, excessive and prolonged inflammatory response to cytokines/chemokines (29). The cytokine storm originates in the lung, and then pro-inflammatory cytokines and chemokines are released from the tissue and circulated to other parts of the body (30). To date, it has been widely accepted that abnormal immune response to SARS-CoV-2 infection is mainly characterized by hyperinflammation, hypercoagulation, and endothelial dysfunction, which are all inter-related with cytokine storm, leading to MODS, such as ARDS and AKI, and subsequent morbidity and mortality (31, 32). Meanwhile, a few patients with critically ill COVID-19 may have mild symptoms in the early stages but suddenly deteriorate or even die in the later stages, further making cytokine storm in the spotlight. Additionally, hemophagocytic lymphohistiocytosis and multisystem inflammatory syndrome associated with SARS-CoV-2 have been recognized as complications due to cytokine storm (33–35). In this regard, controlling the inflammatory response may be as important as targeting the virus in critically ill COVID-19 patients (36). However, the limited understanding of specific inflammatory responses in different pathologies and complex networks of inflammatory responses are insufficient to control the overall inflammatory response. For instance, sepsis-like syndromes may also occur due to viral *per se* or superimposed bacterial infections (37). Unfortunately, microorganisms cannot be identified in up to one-third of the cultures, which will hinder the timely initiation of appropriate antibiotic therapy (38). Meanwhile, irrationally direct use of nonspecific immunomodulators such as corticosteroids can add insult to injury in critically ill COVID-19 patients (39).

Current evidence suggests that conventional blood purification modalities such as dialysis, hemofiltration and plasmapheresis show insufficient performances in removing middle to large cytokine molecules and pathogens (40). Therefore, the rationale for the use of hemoadsorption is indeed strong when specific inflammatory mediators (e.g., cytokines and endotoxin) in critically ill COVID-19 patients with cytokine storm and MODS are selectively targeted, as their reduced levels are associated with decreased morbidity and mortality (18). Because of a lack of well-defined biomarker thresholds to consider the initiation of hemoadsorption, the rate of cytokines removal by hemoadsorption is thought to depend on the high-level of baseline cytokine concentrations in plasma (41), implying that the presence of higher levels of cytokines is associated with a better benefit from cytokine hemoadsorption. Along with a number of novel hemofilters being created in quick succession, hemoadsorption therapy with immunomodulation and toxin clearance is a promising alternative to standard of care in critically ill COVID-19 patients. Considering the interactions of adsorptive hemofilters with pathological mechanisms caused by COVID-19, the potential mechanisms of the effect of hemoadsorption in severe COVID-19 are as follows: 1) reversing the state of immune dysregulation through the elimination of peak cytokine concentrations (42–44); 2) interrupting cascade immune reactions by modulating the composition and kinetic redistribution of mediators in body fluids (45, 46); 3) restoring immune function by regulating monocytes, neutrophils and lymphocytes to increase their sensitivity to drugs and to reduce virus reactivation (47–49); 4) directly eliminating SARS-CoV-2 viral load and pathogen-associated molecular patterns (endotoxins, etc.) (50). Basic characteristics of currently available hemoadsorption therapy in COVID-19 patients are shown in **Table 1**.

**Table 1:** Basic characteristics of currently available hemoadsorption therapy in COVID-19 patients.

Hemofilter (manufacturer)/technique	Composition	Rationale in COVID-19	Reference
oXiris (Baxter International, Deerfield, IL, USA)	Polyethyleneimine with pregrafted heparin layer and negatively charged hydrogel	Adsorption of endotoxin and cytokine, antithrombogenic properties	(51, 52)
CytoSorb (CytoSorbents, Monmouth Junction, NJ, USA)	Highly porous polyvinylpyrrolidone-coated polystyrene-divinylbenzene beads	Non-selective adsorption of inflammatory mediators and toxins	(53, 54)
Seraph 100 Microbind affinity filter (ExThera Medical, Martinez, CA, USA)	Ultra-high molecular weight polyethylene beads with end-point-attached heparin	The reduction of SARS-CoV-2 nucleocapsid protein and RNAemia	(55, 56)
Polymyxin B (Toraymyxin^®^, Toray Medical, Tokyo, Japan)	Polymyxin B-immobilized polypropylene-polystyrene fiber fabrics	Adsorption of endotoxin	(57, 58)
HA resin (Jafron Biomedical Co., China)	Neutro-macroporous resin adsorbing beads made of styrene-divinylbenzene copolymer	Non-selective adsorption of inflammatory mediators and toxins	(59, 60)
ALS	Modules for plasma replacement, plasma adsorption, and blood/plasma filtration	Clearance of inflammatory mediators and small-medium molecule toxins	(61, 62)
SCD (SeaStar Medical, Inc., Denver, CO)	A sequestering membrane and a biologic moiety (citrate)	Clearance of highly activated circulating leukocytes and cytokines	(63, 64)

ALS, Artificial-Liver Blood-Purification System; SCD, selective cytopheretic device.

## 3 Current evidence for hemoadsorption use in severe COVID-19 patients

### 3.1 oXiris membrane

The oXiris membrane (Baxter International, Deerfield, IL, USA) employs a unique polyethyleneimine coating to modify the conventional AN69 membrane, with significant endotoxin and cytokine adsorption properties by the polyethyleneimine layer and the bulk negatively charged hydrogel structures, respectively (51, 65). In addition, the oXiris membrane exhibits antithrombogenic properties due to a pre-grafted heparin layer and has long been used in continuous renal replacement therapy (CRRT) for critically ill patients with sepsis (51, 66). As severe COVID-19 patients frequently develop life-threatening AKI and cytokine storm, the oXiris membrane has been authorized for emergency use in adults with confirmed COVID-19 by the FDA since April 2020 (67).

Most small-size case series (68–71) collectively found that CRRT with the oXiris membrane significantly decreased levels of proinflammatory cytokines and improved hemodynamics and organ function in critically ill COVID-19 patients. A prospective cohort study established the fluctuation of biomarkers over time through the collection of 3,000+ accumulated hours of CRRT with the oXiris hemofilter run-time and real-time data for 44 patients, demonstrating the safety and efficacy of oXiris-CRRT in the reduction of C-reactive protein (CRP) and IL-6 levels, thereby mitigating the systemic damage caused by abnormal immune activation and stabilizing the clinical conditions of participants (46). Compared to the mortality rates calculated by the Acute Physiology and Chronic Health Evaluation (APACHE) IV score, the mean observed mortality rates were also lower after oXiris treatment (69, 71). Premužić et al. further demonstrated that critically ill COVID-19 patients with oXiris treatment survived significantly longer than other intensive care unit (ICU) COVID-19 patients (69). In contrast, a small-size single-center study reported negative results for alleviating cytokine storm in non-AKI patients with severe COVID-19, which might be attributed to the selected non-AKI with normal renal clearance patients and the relatively lower IL-6 concentration (tens of pg/mL) in COVID-19 patients than that in patients with septic shock (72). Furthermore, the differences in inflammatory sub-phenotypes, SARS-CoV-2 viral load, innate and acquired immune defense, and comorbidities may also have a certain impact on the production and release of circulating cytokines and chemokines (72). These findings suggested that routine clinical use of the oXiris membrane in non-AKI COVID-19 patients should be considered with caution.

Although current evidence collectively suggests that the use of the oXiris membrane in COVID-19 patients is well tolerated in most cases, adequate anticoagulation to maintain the patency of the extracorporeal circuit remains a challenge in COVID-19 patients with a hypercoagulable state. Compared to non-COVID-19 patients, a significantly higher incidence of metabolic alkalosis and hypercalcemia consistent with reduced filter patency was observed in COVID-19 patients undergoing CRRT with regional citrate anticoagulation (73). However, the mean half-life of the CRRT hemofilter in COVID-19 patients was similar to that in non-COVID-19 patients with septic shock because hospitalized COVID-19 patients routinely received systemic heparin for thromboprophylaxis (74). These results suggested that close monitoring of the acid-base balance appears warranted when delivering CRRT with regional citrate anticoagulation in severe COVID-19 patients.

Currently, there is an ongoing open-label randomized controlled trial (RCT) (oXAKI-COV study) comparing CRRT with the oXiris membrane vs. standard AN69 membrane during a 72-h treatment period in critically ill COVID-19 patients with AKI (NCT04597034) (75). The primary outcome of the oXAKI-COV study is the change in norepinephrine requirement by at least 0.1 mg/kg/min to maintain similar mean arterial pressure after initiation of CRRT. Secondary outcome measures included the change in interleukin serum levels (IL-6, IL-10, and TNF-α) and length of (intensive care unit) ICU stay in these patients. It is believed that the final analysis of such a high-quality RCT could provide solid evidence in this field and advance clinical practice.

### 3.2 Cytosorb^®^ adsorber

The Cytosorb^®^ (CytoSorbents, Monmouth Junction, NJ, USA) has long been approved for the removal of cytokines, bilirubin, and myoglobin by hemoadsorption (53, 76). The adsorber consists of a cylindrical cartridge filled with tiny, highly porous, hemocompatible polyvinylpyrrolidone-coated polystyrene-divinyl-benzene copolymer beads with a total surface area of > 40,000 m^2^, which significantly adsorbs hydrophobic cytokine molecules within the 5–55 kDa molecular weight range (54, 77). Currently, the Cytosorb^®^ adsorber can be used for hemoadsorption or in series with CRRT and extracorporeal membrane oxygenation (ECMO) circuits (43, 78, 79), and the duration of Cytosorb therapy usually permits at least 72 continuous hours with device exchange every 24 hours (78). Currently, the Cytosorb^®^ adsorber has been broadly used in patients with critical illnesses such as infective endocarditis (80, 81), severe acute pancreatitis (82), postcardiac arrest syndrome (83), and septic shock (84) during the last decade. Early in the COVID-19 pandemic, extracorporeal hemoadsorption with the Cytosorb^®^ adsorber was also approved as an adjunctive therapy to remove excessive inflammatory mediators in COVID-19 patients by the European Union and FDA.

Data on clinical effectiveness are inconsistent. Most small-size observational studies or case series consistently found that CytoSorb treatment was effective in alleviating inflammation [IL-6, procalcitonin, CRP, ferritin] (85–89), decreasing D-dimer (86), and improving oxygenation and hemodynamics (88–90) in critically ill COVID-19 patients with refractory ARDS or MODS. In a case series study enrolling 6 COVID-19 patients who were characterized by severe acute respiratory failure with poor response to the prone position (PaO_2_/FiO_2_ [arterial oxygen pressure (PaO_2_), inspired fraction of oxygen (FiO_2_)] ratio remained <150 after the prone position) and hyperinflammatory state (IL-6 > 1,000 pg/ml and increased levels of ferritin and D-dimer), cytokine hemoadsorption with the CytoSorb adsorber was used as an effective and safe rescue therapy. After the CytoSorb treatment, the extra high median baseline IL-6 concentration (17,367 pg/ml [4,539–22,532]) had a significant reduction to 2.403 pg/ml [917–3.724], p = 0.043. Oxygenation also improved significantly from 103 (18.4) mm Hg to 222 (20.9) mm Hg, p = 0.029 (86). A multicenter, retrospective registry enrolling 52 patients who received veno-venous ECMO plus CytoSorb therapy at 5 medical centers in the USA also demonstrated that CytoSorb therapy was associated with lower 90-day in-hospital mortality (26.9%) than the ELSO ECMO COVID-19 Registry (52%), suggesting the potential survival benefit of cytokine adsorption (78). Moreover, CytoSorb therapy was well tolerated without any device-related adverse events reported (78, 79).

Disappointingly, data from three RCTs investigating the effect of CytoSorb^®^ in COVID-19 patients showed inconsistent findings (43, 79, 91). In the CYCOV study, Supady et al. found a significantly higher mortality in 14 of 17 COVID-19 patients (82%) receiving ECMO and CytoSorb therapy compared with 4 of 17 ECMO patients (24%) treated without cytokine adsorption (43). There was also no significant difference for IL-6 between the two groups after 72 h of ECMO, which might be attributed to low median baseline IL-6 levels (357 ng/L) in the intervention group. In contrast, the data from the international CytoSorb registry suggests that serum IL-6 concentrations can be reduced from a median of 5000 pg/mL down to 289 pg/mL after 24 h of cytokine adsorption in severe patients, suggesting that COVID-19 patients with higher levels of cytokines might benefit more from cytokine hemoadsorption with the Cytosorb treatment (41, 92). In another prospective, randomized controlled pilot study, 23 COVID-19 patients with vasoplegic shock and MODS were randomized to receive Cytosorb^®^ therapy incorporated in the continuous veno-venous hemodiafiltration (CVVHD) circuit, and 26 patients received standard CVVHD therapy (79). The results showed that hemoadsorption with Cytosorb^®^ did not decrease the time until resolution of vasoplegic shock (5 d, interquartile range: 4-5 d) compared with the control group (4 d, interquartile range: 3-5 d). Importantly, the ICU mortality rate was 78% in the CytoSorb^®^ group and 73% in the control group (unadjusted hazard ratio, 1.17 [95% CI, 0.61-2.23]; p=0.64). Meanwhile, the effects on the kinetics of inflammatory parameters (e.g., IL-6 and CRP) and catecholamine requirements were similar between the groups. The negative results may be attributed to the late intervention given the severity of disease, and the results of the statistical analysis were limited by the total number of cases. In addition, it was unclear to what extent vasoparalytic shock can be attributed to COVID-19-driven hyperinflammation or sepsis due to secondary recurrent infections, so there might be potential confounding factors. In the latest prospective, randomized controlled pilot study to date, 24 COVID-19 patients with refractory shock, hypercytokinemia (defined as IL-6 ≥500 ng/L), and indication for RRT or ECMO were enrolled. Compared with standard of care, hemoadsorption with the CytoSorb^®^ adsorber for up to 5 days was not associated with an significant improvement in shock resolution (33% vs. 17%, p=0.640) and survival (42% vs. 33%, p=1.0), possibly because critically ill patients with high sequential organ failure assessment (SOFA) and simplified acute physiology score II scores in this cohort were more likely to have a high in-hospital mortality (91). Altogether, the inconsistent results from RCTs call for a very careful application of Cytosorb^®^ in severe COVID-19 patients requiring ECMO or RRT. The indication and optimal initiation timing for CytoSorb treatment should also be investigated in future high-quality RCTs.

### 3.3 Seraph 100 Microbind affinity filter

A Seraph 100 Microbind affinity blood filter (ExThera Medical, Martinez, CA, USA) is an extracorporeal heparin-immobilized sorbent hemofilter that can remove pathogens from the bloodstream. On April 17th, 2020, the FDA granted COVID-19 emergency use authorizations for the Seraph 100 filter because it can utilize the structural similarity between heparin and pathogen receptors (e.g., heparan sulphate) to bind certain pathogens (55, 93). Heparin binding of the spike protein is of more clinical significance in SARS-CoV-2 than in other coronaviruses because viral RNAemia is more frequent (up to 78%) in critically ill COVID-19 patients (94, 95) and is associated with COVID-19 severity. In a small-size case series, Seraph 100 was found to decrease SARS-CoV-2 nucleocapsid protein and RNAemia/viraemia in the blood of critically ill COVID-19 patients (56).

Several cases reported that treatment with Seraph 100 was associated with a rapid improvement in oxygenation (96) and reduced D-dimers (97), and most cases behaved well tolerated and had a good clinical response (98, 99). The latest interim analysis of the COSA registry enrolling 78 COVID-19 patients showed that the observed 30-day mortality rate in the registry was lower than the mortality predicted by the coronavirus clinical characterization consortium score (11.1% vs. 38.0%) in non-ICU patients and the sequential organ failure assessment score (50.7% vs. 56.7%) in ICU patients (100). Although more than half of the treatments were performed in conjunction with renal replacement therapy, the premature end of treatment due to circuit failure was reported in 9 (8.8%) of the 102 treatments with Seraph 100, which was less likely to occur than CRRT sessions in COVID-19 patients (101). Moreover, multivariate Cox regression revealed that delayed Seraph^®^ 100 treatment after ICU admission (>60 h) was associated with increased mortality (100). Most recently, the PURIFY-OBS-1 study included 53 COVID-19 patients treated with Seraph 100 and another 53 matched control patients in 9 participating ICUs across the USA. The Seraph 100 group had lower Charlson comorbidity index scores and APACHE II scores with higher vasopressor-free days than the control group. On univariate analysis, Seraph 100 treatment was associated with decreased mortality with an odds ratio of 0.26 (95% CI: 0.12–0.59). However, a survival benefit with Seraph 100 treatment compared with the external Penn Medicine cohort was not observed in a *post hoc* analysis (102).

Currently, an RCT (NCT04547257) evaluating the safety and effectiveness of Seraph 100 in COVID-19 patients with organ dysfunction is ongoing in Germany and Spain, which takes the change in organ failure from baseline to 48 hours as a primary outcome (103). All-cause 28-day mortality, organ dysfunction-free days, and reduction of viral load will be used as secondary outcome measures. This study is estimated to be completed by the end of 2022.

### 3.4 Polymyxin B hemoperfusion

The Polymyxin B hemoperfusion column (Toraymyxin^®^, Toray Medical, Tokyo, Japan) is composed of polymyxin B-immobilized polypropylene-polystyrene fiber fabrics. Polymyxin B hemoperfusion (PMX) is characterized by removing endotoxin for the treatment of sepsis caused by gram-negative bacteria (18). Recently, common multidrug-resistant bacterial infection in COVID-19 patients has brought it back into our sight (104), and PMX has been suggested to alleviate the peak of endotoxins in COVID-19 patients with secondary bacterial infection, thereby restoring immune homeostasis without prolonging the immunosuppressed state (105, 106). The latest approval of Canada on the use of Toraymyxin^®^ in severe COVID-19 was announced on April 20, 2020 (107). Besides removal of endotoxins, other possible mechanisms for Toraymyxin^®^ use in COVID-19 including cytokine regulation, removal of activated neutrophils, and prevention of the migration of activated leukocytes to the lungs deserve further exploration (57, 58). As the only direct hemoperfusion device targeting endotoxin, PMX can be intermittently performed without dialysis (108).

Early case reports showed an improvement in PaO_2_/FiO_2_ (109, 110) and a reduction in serum CRP levels after Toraymyxin^®^ treatment (111). Likewise, Mayuko et al. reported that Toraymyxin^®^ treatment decreased inflammatory markers and improved oxygenation in a COVID-19 patient with respiratory failure and hyperinflammation, which halted the patient’s progression to ARDS and avoided the need for mechanical ventilation (112). In another case series, Daisuke et al. performed 22 PMX sessions on 12 COVID-19 patients with a PaO_2_/FiO_2_ < 300 (113). On day 14 after the first Toraymyxin^®^ treatment, disease severity decreased in 7 of 12 patients, with an increased PaO_2_/FiO_2_ ratio and decreased urine β2-microglobulin. In addition, cytokine measurements before and after Toraymyxin^®^ treatment revealed decreased IL-6 levels. However, coagulation-related events still occurred in 12 of the 22 cases (54.5%) during the course of treatment, causing the need for reconfiguration of the circuit. It is still difficult to determine whether longer (>12 hours) treatment with PMX is effective in improving oxygenation (113). In a recent case series study from EUPHAS2 registry, PMX treatment was also used in 12 COVID-19 patients with sepsis. The results showed that SOFA score progressively improved after 120 hours of PMX treatment, along with a significant decrease of median endotoxin activity assay (EAA) from 0.78 [0.70-0.92] to 0.60 [0.44-0.72], suggesting that the measurement of contemporary EAA levels can be used for therapeutical efficacy monitoring during treatment (114).

### 3.5 HA resin hemoperfusion cartridges

HA resin hemoperfusion cartridges (HA130, HA230, HA330 and HA380) (Jafron Biomedical Co., China) have been widely used to remove a wide spectrum of endogenous and exogenous toxins (59). The cartridges contain highly biocompatible neutro-macroporous resin adsorbing beads made of styrene-divinylbenzene copolymer and have a high surface area (115). In acute inflammatory conditions such as sepsis, acute lung injury, hepatitis, and pancreatitis, HA 330 and HA 380 cartridges significantly remove excessive proinflammatory cytokines (IL-6, IL-10, TNF-α) in the bloodstream (59, 116). The recommended treatment duration of HA330 and HA380 cartridges is usually 2 to 2.5 h.

A prospective cohort study in Thailand compared the efficacy of additional hemoperfusion with standard of care on 29 severe COVID-19 patients admitted to the ICU (117). Patients who received at least 3 sessions of HA 330 hemoperfusion therapy were defined as the hemoperfusion group, while those who were treated by standard of care alone or received less than 3 sessions of HA-330 hemoperfusion were classified as the control group. Compared to the control group, patients in the hemoperfusion group showed a clinical improvement associated with a decreased SOFA score, and the addition of at least 3 sessions of HA330 hemoperfusion to standard treatment could alleviate organ failure and reduce mortality (117). However, only serum CRP levels in the patients were monitored to evaluate the effect of HA330 hemoperfusion on cytokine removal. Another single-center, matched control retrospective study enrolled 128 COVID-19 patients to investigate the efficacy of hemoperfusion in combination with standard therapy in critically ill COVID-19 patients (118). Of 55 patients in the hemoperfusion group, the number of patients who received one, two, and three or four courses of hemoperfusion was 18 (32.7%), 14 (25.4%), and 23 (41.9%), respectively. The results showed that the mortality rate was significantly lower in the HA 330 hemoperfusion group than in the matched group (67.3% vs. 89%, p= 0.002). In addition, the median length of ICU stay, duration of incubation, and median final SPO_2_ were significantly higher in the hemoperfusion group than in the matched group. Likewise, Ruslan et al. demonstrated that cytokine adsorption with HA330 or Mediasorb cartridges significantly decreased CRP and fibrinogen at postfiltration in COVID-19 patients admitted to the ICU (119). However, there was no improvement in patient-centered outcomes such as SOFA scores, vasopressor use and in-hospital mortality.

Extracorporeal hemoperfusion therapies with HA resin cartridges are also associated with a number of complications, such as hematomas at insertion sites, pneumothorax, infections, and nonselective removal of nutrients and drugs (120, 121). Consequently, it is crucial to consider drug elimination during HA resin cartridge hemoadsorption sessions. The optimal timing for hemoadsorption administration in critically ill patients with COVID-19 should also be further determined in the future.

### 3.6 Artificial liver blood purification system

The Artificial-Liver Blood-Purification System (ALS) integrates plasma exchange, hemoperfusion, continuous hemofiltration, hemodialysis and bilirubin adsorption (61). It has been well established that ALS is effective in eliminating inflammatory mediators and small-medium molecules to maintain water/electrolyte balance and homeostasis (122). Despite the lack of solid evidence, the use of ALS in severe COVID-19 patients with cytokine storm was recommended by a Chinese expert consensus in early 2020 (122). Subsequently, through a paired study analyzing serum cytokine levels pre- and post-ALS, a nonrandomized clinical trial found that three consecutive courses of ALS treatment significantly decreased the plasma levels of 32 cytokines, including IL-6 and TNF-α (44). Furthermore, the APACHE II and SOFA scores also decreased after three consecutive sessions with ALS (44). Another case series consistently showed that the levels of IL-6 and IL-10 significantly declined after treatment with ALS (123). More recently, a multicenter, prospective study enrolling 101 participants found that, beyond a remarkable reduction in plasma IL-6 concentration, the 28-day mortality of COVID patients in the ALS group (16%) was significantly lower than that of the control group (50.98%), suggesting that ALS treatment could block cytokine storm and reduce short-term mortality (61). However, given the complexity of the modules of ALS, more detailed studies on the mechanism and long-term follow-up are needed.

### 3.7 Selective cytopheretic device

The selective cytopheretic device (SCD) is an immunomodulatory extracorporeal device that can promote a lower proinflammatory phenotype in circulating neutrophils and monocytes, thereby modulating the immune response and moderating tissue damage (63, 124). The device is usually placed postfilter in the CRRT circuit with regional citrate anticoagulation to facilitate leukocyte binding to the filter (125). An early case report showed that treatment with SCD in two COVID-19 patients with severe ARDS resulted in significant reductions in inflammatory markers, including procalcitonin, D-dimer, LDH, ferritin, CRP, and IL-6 (63). The PaO_2_/FiO_2_ ratios of the two enrolled patients also increased from 55 and 58 to 200 and 192, respectively, within hours of SCD initiation. Another recent prospective, single-arm treatment clinical trial at two academic medical centers enrolled 22 COVID-10 patients with ARDS to further evaluate the safety and clinical outcomes of extracorporeal immunomodulation treatment with SCD (49). The results of flow cytometry demonstrated that SCD selectively eliminated highly activated circulating leukocytes and diminished the inflammatory phenotype of circulating effector cells, with significant reductions in plasma levels of proinflammatory cytokines, including IL-6, IL-15, and soluble ST2 (49). More importantly, the mortality rate of the patients who received greater than 96 hours of SCD treatment was significantly lower than that of a contemporaneous control (31% vs. 81%, p < 0.012) (49). These encouraging findings suggested that early intervention with SCD in critically ill COVID-19 patients might be associated with an improvement in systemic inflammation and a potential survival benefit. It is also noteworthy that no device-related serious adverse events were observed during extracorporeal SCD sessions. A summary of prospective studies evaluating hemoadsorption in COVID-19 patients are also shown in **Table 2**.

**Table 2:** Summary of prospective studies evaluating hemoadsorption in COVID-19 patients.

Study design	First Author, year, Country	Hemofilter	Population/Sample size	Levels of cytokines	Trial design/Intervention	Primary outcome/Time of assessment	Main findings	Limitations
RCT	Jarczak, 2022, Germany (91)	CytoSorb	COVID-19 patients with refractory shock, hypercytokinemia (IL-6 ≥500 ng/L), and indication for RRT or ECMO, n=24	IL-6 levels were 2,269 (948–3,679) ng/L in the CytoSorb group and 3,747 (1,301-5,415) ng/L in the standard of care group (p= 0.378)	Hemoperfusion with CytoSorb for up to 5 days vs. standard of care	Hemodynamic improvement (norepinephrine ≤0.05 µg/kg/min ≥24 h)	Compared with standard of care, HP with CytoSorb was associated with an insignificant improvement in shock resolution (33% vs. 17%, p=0.640) and survival (42% vs. 33%, p=1.0).	- Differences regarding age and norepinephrine dose at baseline between both groups - Inherent bias of trials involving rather complex medical devices - Whether longer duration or an earlier start of HP with CytoSorb would result in an improved outcome remains unclear.
RCT	Stockmann, 2022, Germany (79)	CytoSorb	COVID-19 patients with vasoplegic shock, hyperinflammation (CRP value greater than 100 mg/L), and indication for hemodialysis, n=23	IL-6 levels were 591.0 (23.9–1,852.8), ng/L in the CytoSorb group and 552.5 (299.5–1,787.5) ng/L in the control group	Hemoperfusion with CytoSorb for 3-7 days vs. standard therapy	Time until resolution of vasoplegic shock (no need for vasopressors for at least 8 h to sustain an MAP greater than or equal to 65 mm Hg)	Resolution of vasoplegic shock was observed in 13 of 23 patients (56.5%) in the CytoSorb and 12 of 26 patients (46.2%) in the control group, and the HR was 1.23 (95% CI, 0.54-2.79); p = 0.63.	- Without formal sample size calculation - Whether an earlier start of HP with CytoSorb would result in an improved outcome remains unclear given the severity of disease with vasoplegic shock and multiple organ failure. - Potential confounding factors
RCT	Supady, 2021, Germany (43)	CytoSorb	Severe COVID-19 patients requiring ECMO, n=34	Median baseline IL-6 levels was 357 ng/L	Hemoperfusion with CytoSorb for 72 h	Serum IL-6 concentration after treatment with hemoperfusion	Adjusted mean log IL-6 concentrations after 72 h were 0.30 higher in the cytokine adsorption group (95% CI, 0·70, 1·30, p=0·54). Survival after 30 days was three (18%) of 17 with cytokine adsorption and 13 (76%) of 17 without cytokine adsorption (p=0·0016).	- The large variability of the degree of systemic inflammation in patient cohort (different baseline concentrations for IL-6.) - Small sample size does not allow meaningful sub-group analyses - Inferences about cytokine adsorption for shorter or longer periods during ECMO support in COVID-19 nor about cytokine adsorption at different timepoints during the course of the disease was not allowed
A prospective cohort study	Rosalia, 2022, Italy (46)	oXiris	COVID-19 patients, n=44	IL-6 levels was 15.5 (7.4–47.3) pg/mL	Treatment with oXiris membrane for immunomodulation and support to renal function during AKI	The overtime variation of inflammatory biomarkers	Treatment with oXiris was associated with a decrease in CRP, and control of IL-6 and procalcitoni.	- Observational design, the absence of randomization and limited cohort size
A prospective cohort study	Surasit, 2022, Thailand (117)	HA-330	Severe COVID-19, hypoxemic respiratory failure with PaO_2_ to FiO_2_ ratio < 200, systemic inflammation (CRP≥ 30 mg/L), n=29	CRP values were 96.79 (65, 197) mg/L in the hemoperfusion group and 87.3 (37.6, 185.6) mg/L in the control group (p= 0.53)	The addition of at least 3 sessions of HA-330 hemoperfusion to standard therapy	Daily SOFA scores	Completion of at least 3 sessions of HA-330 hemoperfusion therapy was associated with decreased SOFA score (Adj. β-coefficient = −1.28; p = 0.008).	- Limited sample size - The prospective study with regression analysis still has unavoidable bias and confounders - The IL-6 level was not checked - The optimal session of hemoperfusion is still unclear
A nonrandomized clinical trial	Guo J, 2020, China (44)	ALS	Critically ill COVID-19 patients in single-center, n=12	NA (serum cytokine levels pre- and post-ALS analyzed through a paired study)	Three consecutive courses of ALS therapy	The levels of cytokines pre- and post-ALS	A total of 32 cytokines were found to be significantly decreased after ALS therapy.	- The nonrandomized clinical trial with limited sample size has unavoidable bias and confounders
A two-center, prospective, single-arm treatment clinical trial	Yessayan, 2022, USA (49)	SCD	COVID-19 patients in the ICU with ARDS who required mechanical ventilation, n=22	NA	Treatment with an SCD integrated into a CRRT circuit for up to 10 days	Mortality rate	SCD-treated subjects had a reduction in 60-day mortality of 50% compared with 81% in the control cohort. The subjects who received greater than 96 hours of SCD treatment, per protocol, had a further reduction in mortality to 31% (p < 0.012).	- The control group was a nonrandomized contemporaneous control group at each clinical site - The modest data set in the control group for comparisons with the treated group - Potential bias due to different preferences for duration of life support and withdrawal of care

RCT, randomized controlled trial; RRT, renal replacement therapy; ECMO, extracorporeal membrane oxygenation; MAP, mean arterial pressure; HR, hazard ratio; IL-6, interleukin-6; AKI, acute kidney injury; CRP, C-reactive protein; SOFA, sequential organ failure assessment; ALS, Artificial-Liver Blood-Purification System; SCD, selective cytopheretic device; ICU, intensive care unit; ARDS, acute respiratory distress syndrome; CRRT, continuous renal replacement therapy.

## 4 Summary and future perspectives

Although the management of critically ill COVID-19 patients is still challenging, hemoadsorption therapy may be life-saving by regulating immune homeostasis, alleviating viraemia, and reducing endotoxin activity in critically ill COVID-19 patients. Inspiringly, data from low-quality case series and observational studies show that hemoadsorption therapy effectively reduces the levels of inflammatory mediators and improves hemodynamics and organ function. However, it is worth noting that there are still several open problems to tackle: 1) knowledge of unintended removal during hemoadsorption is still scant, and the determination of an individualized anticoagulation regime remains a puzzle that is much more complicated due to the hypercoagulable state in COVID-19 patients; 2) the optimal timing of initiation and duration of hemoadsorption and hemofilter replacement intervals are unknown; 3) the identification of specific patient subgroup who will benefit from hemoadsorption therapy is urgently required; 4) the effect of hemoadsorption therapy in patient-centered outcomes remains to be investigated. Therefore, for the moment these techniques should be considered experimental, high-quality of clinical studies with standardized study design and implementation, rigorous quality control, individualized consideration of risks and benefits, and even evidence-based advice on health economics in such resource-constrained settings are still needed.

## Author contributions

KC, YL, and BS conceived the idea, KC and YL performed the literature search and drafted the manuscript. The article was critically reviewed and revised by all authors. All authors contributed to the article and approved the submitted version.

## References

[1] COVID-19 Excess Mortality Collaborators . Estimating excess mortality due to the COVID-19 pandemic: A systematic analysis of COVID-19-related mortality, 2020-21. Lancet (London England) (2022) 399(10334):1513–1536. doi: 10.1016/S0140-6736(21)02796-3 PMC8912932 35279232

[2] Ekaney ML Otto GP Sossdorf M Sponholz C Boehringer M Loesche W . Impact of plasma histones in human sepsis and their contribution to cellular injury and inflammation. Crit Care (2014) 18(5):543. doi: 10.1186/s13054-014-0543-8 25260379 PMC4201918

[3] Murakami N Hayden R Hills T Al-Samkari H Casey J Del Sorbo L . Therapeutic advances in COVID-19. Nat Rev Nephrol (2022) 19(1):38–52. doi: 10.1038/s41581-022-00642-4 PMC9574806 36253508

[4] Mohammed I Nauman A Paul P Ganesan S Chen KH Jalil SMS . The efficacy and effectiveness of the COVID-19 vaccines in reducing infection, severity, hospitalization, and mortality: A systematic review. Hum Vaccines immunotherapeutics (2022) 18(1):2027160. doi: 10.1080/21645515.2022.2027160 PMC8862168 35113777

[5] Al-Kassim Hassan M Adam Bala A Jatau AI . Low rate of COVID-19 vaccination in Africa: A cause for concern. Ther Adv Vaccines Immunother (2022) 10: 25151355221088159. doi: 10.1177/25151355221088159 35355936 PMC8958672

[6] Gottlieb RL Vaca CE Paredes R Mera J Webb BJ Perez G . Early remdesivir to prevent progression to severe covid-19 in outpatients. N Engl J Med (2022) 386(4):305–15. doi: 10.1056/NEJMoa2116846 PMC8757570 34937145

[7] Ely EW Ramanan AV Kartman CE de Bono S Liao R Piruzeli MLB . Efficacy and safety of baricitinib plus standard of care for the treatment of critically ill hospitalised adults with COVID-19 on invasive mechanical ventilation or extracorporeal membrane oxygenation: An exploratory, randomised, placebo-controlled trial. Lancet Respir Med (2022) 10(4):327–36. doi: 10.1016/S2213-2600(22)00006-6 PMC8813065 35123660

[8] Marconi VC Ramanan AV de Bono S Kartman CE Krishnan V Liao R . Efficacy and safety of baricitinib for the treatment of hospitalised adults with COVID-19 (COV-BARRIER): A randomised, double-blind, parallel-group, placebo-controlled phase 3 trial. Lancet Respir Med (2021) 9(12):1407–18. doi: 10.1016/S2213-2600(21)00331-3 PMC8409066 34480861

[9] Welker J Pulido JD Catanzaro AT Malvestutto CD Li Z Cohen JB . Efficacy and safety of CD24Fc in hospitalised patients with COVID-19: A randomised, double-blind, placebo-controlled, phase 3 study. Lancet Infect diseases (2022) 22(5):611–21. doi: 10.1016/S1473-3099(22)00058-5 PMC8916779 35286843

[10] Karim SSA Karim QA . Omicron SARS-CoV-2 variant: A new chapter in the COVID-19 pandemic. Lancet (London England) (2021) 398(10317):2126–8. doi: 10.1016/S0140-6736(21)02758-6 PMC8640673 34871545

[11] Gu X Cao B . Understanding of COVID-19 from infection-fatality ratio. Lancet (London England) (2022) 399(10334):1442–3. doi: 10.1016/S0140-6736(22)00281-1 PMC8871411 35219375

[12] Zhu Y Chen X Liu X . NETosis and neutrophil extracellular traps in COVID-19: Immunothrombosis and beyond. Front Immunol (2022) 13:838011. doi: 10.3389/fimmu.2022.838011 35309344 PMC8924116

[13] Wendel-Garcia PD Moser A Jeitziner MM Aguirre-Bermeo H Arias-Sanchez P Apolo J . Dynamics of disease characteristics and clinical management of critically ill COVID-19 patients over the time course of the pandemic: An analysis of the prospective, international, multicentre RISC-19-ICU registry. Crit Care (London England) (2022) 26(1):199. doi: 10.1186/s13054-022-04065-2 PMC9254551 35787726

[14] RECOVERY Collaborative Group . Aspirin in patients admitted to hospital with COVID-19 (RECOVERY): A randomised, controlled, open-label, platform trial. Lancet (London England) (2022) 399(10320):143–51. doi: 10.1016/S0140-6736(21)01825-0 PMC8598213 34800427

[15] Bradbury CA Lawler PR Stanworth SJ McVerry BJ McQuilten Z Higgins AM . Effect of antiplatelet therapy on survival and organ support-free days in critically ill patients with COVID-19: A randomized clinical trial. Jama (2022) 327(13):1247–59. doi: 10.1001/jama.2022.2910 PMC8941448 35315874

[16] De Santis GC Oliveira LC Garibaldi PMM Almado CEL Croda J Arcanjo GGA . High-dose convalescent plasma for treatment of severe COVID-19. Emerging Infect diseases (2022) 28(3):548–55. doi: 10.3201/eid2803.212299 PMC8888205 35081022

[17] Declercq J Van Damme KFA De Leeuw E Maes B Bosteels C Tavernier SJ . Effect of anti-interleukin drugs in patients with COVID-19 and signs of cytokine release syndrome (COV-AID): A factorial, randomised, controlled trial. Lancet Respir Med (2021) 9(12):1427–38. doi: 10.1016/S2213-2600(21)00377-5 PMC8555973 34756178

[18] Ronco C Bellomo R . Hemoperfusion: technical aspects and state of the art. Crit Care (London England) (2022) 26(1):135. doi: 10.1186/s13054-022-04009-w PMC9097563 35549999

[19] Supady A Brodie D Wengenmayer T . Extracorporeal haemoadsorption: does the evidence support its routine use in critical care? Lancet Respir Med (2022) 10(3):307–12. doi: 10.1016/S2213-2600(21)00451-3 34921755

[20] Ricci Z Romagnoli S Reis T Bellomo R Ronco C . Hemoperfusion in the intensive care unit. Intensive Care Med (2022) 48(10):1397–408. doi: 10.1007/s00134-022-06810-1 PMC9389493 35984473

[21] Sanfilippo F Martucci G La Via L Cuttone G Dimarco G Pulizzi C . Hemoperfusion and blood purification strategies in patients with COVID-19: A systematic review. Artif Organs (2021) 45(12):1466–76. doi: 10.1111/aor.14078 PMC8652899 34632596

[22] Koc S Uysal H . Literature review of hemadsorption therapy in severe COVID-19 cases: A narrative review. Clin Lab (2022) 68(2):10.7754. doi: 10.7754/Clin.Lab.2021.210839 35142202

[23] Safari S Salimi A Zali A Jahangirifard A Bastanhagh E Aminnejad R . Extracorporeal hemoperfusion as a potential therapeutic option for severe COVID-19 patients; a narrative review. Arch Acad Emerg Med (2020) 8(1):e67. 33134963 PMC7587998

[24] Chen G Zhou Y Ma J Xia P Qin Y Li X . Is there a role for blood purification therapies targeting cytokine storm syndrome in critically severe COVID-19 patients? Renal failure (2020) 42(1):483–8. doi: 10.1080/0886022X.2020.1764369 PMC7946020 32438839

[25] Sette A Crotty S . Adaptive immunity to SARS-CoV-2 and COVID-19. Cell (2021) 184(4):861–80. doi: 10.1016/j.cell.2021.01.007 PMC7803150 33497610

[26] Karki R Kanneganti TD . Innate immunity, cytokine storm, and inflammatory cell death in COVID-19. J Trans Med (2022) 20(1):542. doi: 10.1186/s12967-022-03767-z PMC9682745 36419185

[27] Kasuga Y Zhu B Jang KJ Yoo JS . Innate immune sensing of coronavirus and viral evasion strategies. Exp Mol Med (2021) 53(5):723–36. doi: 10.1038/s12276-021-00602-1 PMC8099713 33953325

[28] Mathew D Giles JR Baxter AE Oldridge DA Greenplate AR Wu JE . Deep immune profiling of COVID-19 patients reveals distinct immunotypes with therapeutic implications. Sci (New York NY) (2020) 369(6508): eabc8511 doi: 10.1126/science.abc8511. PMC7402624 32669297

[29] Ramasamy S Subbian S . Critical determinants of cytokine storm and type I interferon response in COVID-19 pathogenesis. Clin Microbiol Rev (2021) 34(3): e00299-20. doi: 10.1128/CMR.00299-20 33980688 PMC8142516

[30] Alam MS Czajkowsky DM . SARS-CoV-2 infection and oxidative stress: Pathophysiological insight into thrombosis and therapeutic opportunities. Cytokine Growth factor Rev (2022) 63:44–57. doi: 10.1016/j.cytogfr.2021.11.001 34836751 PMC8591899

[31] Prasad M Leon M Lerman LO Lerman A . Viral endothelial dysfunction: A unifying mechanism for COVID-19. Mayo Clinic Proc (2021) 96(12):3099–108. doi: 10.1016/j.mayocp.2021.06.027 PMC8373818 34863398

[32] Merad M Martin JC . Pathological inflammation in patients with COVID-19: A key role for monocytes and macrophages. Nat Rev Immunol (2020) 20(6):355–62. doi: 10.1038/s41577-020-0331-4 PMC7201395 32376901

[33] Mehta Y Dixit SB Zirpe KG Ansari AS . Cytokine storm in novel coronavirus disease (COVID-19): Expert management considerations. Indian J Crit Care Med (2020) 24(6):429–34. doi: 10.5005/jp-journals-10071-23415 PMC7435090 32863636

[34] Mahdavi M Hejri GM Pouraliakbar H Shahzadi H Hesami M Houshmand G . Cytokine storm after heart transplantation in COVID-19-related haemophagocytic lymphohistiocytosis (HLH). ESC Heart failure (2022) 9(1):219–23. doi: 10.1002/ehf2.13728 PMC8788054 34821079

[35] Lalwani P Baskaran S Uribe DA Ramaiah A Saqib A ElMesserey M . A case of COVID-19-Associated pediatric multisystem inflammatory syndrome in shock managed by cytokine filtration. Case Rep Pediatrics (2022) 2022:3373289. doi: 10.1155/2022/3373289 PMC8808292 35127192

[36] Tay MZ Poh CM Rénia L MacAry PA Ng LFP . The trinity of COVID-19: immunity, inflammation and intervention. Nat Rev Immunol (2020) 20(6):363–74. doi: 10.1038/s41577-020-0311-8 PMC7187672 32346093

[37] Pancani F Pavani R Quacquarelli A Feri M . Successful use of CytoSorb in a covid-19 patient with secondary septic shock due to a sacral decubitus infection. Int J Artif Organs (2021) 44(12):1034–8. doi: 10.1177/03913988211016473 33998306

[38] Cascarano L Cutuli SL Pintaudi G Tanzarella ES Carelli S Anzellotti G . Extracorporeal immune modulation in COVID-19 induced immune dysfunction and secondary infections: the role of oXiris® membrane. Minerva Anestesiologica (2021) 87(3):384–5. doi: 10.23736/S0375-9393.20.15124-1 33331748

[39] Lim MX Fong KK Yeap TB . Use of extracorporeal blood purification therapy (ECBPT) as an adjuvant to high-dose corticosteroids in a severely ill COVID-19 patient with concomitant bacterial infection. BMJ Case Rep (2021) 14(10): e245639. doi: 10.1136/bcr-2021-245639 PMC8515465 34645636

[40] Wolley M Jardine M Hutchison CA . Exploring the clinical relevance of providing increased removal of Large middle molecules. Clin J Am Soc Nephrol CJASN (2018) 13(5):805–14. doi: 10.2215/CJN.10110917 PMC5969479 29507008

[41] Kellum JA Kong L Fink MP Weissfeld LA Yealy DM Pinsky MR . Understanding the inflammatory cytokine response in pneumonia and sepsis: results of the genetic and inflammatory markers of sepsis (GenIMS) study. Arch Internal Med (2007) 167(15):1655–63. doi: 10.1001/archinte.167.15.1655 PMC4495652 17698689

[42] Ronco C Bellomo R . Acute renal failure and multiple organ dysfunction in the ICU: from renal replacement therapy (RRT) to multiple organ support therapy (MOST). Int J Artif Organs (2002) 25(8):733–47. doi: 10.1177/039139880202500801 12296458

[43] Supady A Weber E Rieder M Lother A Niklaus T Zahn T . Cytokine adsorption in patients with severe COVID-19 pneumonia requiring extracorporeal membrane oxygenation (CYCOV): A single centre, open-label, randomised, controlled trial. Lancet Respir Med (2021) 9(7):755–62. doi: 10.1016/S2213-2600(21)00177-6 PMC8121541 34000236

[44] Guo J Xia H Wang S Yu L Zhang H Chen J . The artificial-liver blood-purification system can effectively improve hypercytokinemia for COVID-19. Front Immunol (2020) 11:586073. doi: 10.3389/fimmu.2020.586073 33424838 PMC7786016

[45] Honore PM Joannes-Boyau O . High volume hemofiltration (HVHF) in sepsis: A comprehensive review of rationale, clinical applicability, potential indications and recommendations for future research. Int J Artif Organs (2004) 27(12):1077–82. doi: 10.1177/039139880402701211 15645619

[46] Rosalia RA Ugurov P Neziri D Despotovska S Kostoska E Veljanovska-Kiridjievska L . Extracorporeal blood purification in moderate and severe COVID-19 patients: A prospective cohort study. Blood Purification (2022) 51(3):233–42. doi: 10.1159/000515627 PMC8339014 34126617

[47] Peng Z Singbartl K Simon P Rimmelé T Bishop J Clermont G . Blood purification in sepsis: A new paradigm. Contrib Nephrol (2010) 165:322–8. doi: 10.1159/000313773 20427984

[48] Lancman G Mascarenhas J Bar-Natan M . Severe COVID-19 virus reactivation following treatment for b cell acute lymphoblastic leukemia. J Hematol Oncol (2020) 13(1):131. doi: 10.1186/s13045-020-00968-1 33008453 PMC7531062

[49] Yessayan LT Neyra JA Westover AJ Szamosfalvi B Humes HD . Extracorporeal immunomodulation treatment and clinical outcomes in ICU COVID-19 patients. Crit Care Explorations (2022) 4(5):e0694. doi: 10.1097/CCE.0000000000000694 PMC9126516 35620768

[50] Tampe D Korsten P Bremer SCB Winkler MS Tampe B . Kinetics of bilirubin and ammonia elimination during hemadsorption therapy in secondary sclerosing cholangitis following ECMO therapy and severe COVID-19. Biomedicines (2021) 9(12): 1841. doi: 10.3390/biomedicines9121841 34944657 PMC8698542

[51] Guan M Wang H Tang X Zhao Y Wang F Zhang L . Continuous renal replacement therapy with adsorbing filter oXiris in acute kidney injury with septic shock: A retrospective observational study. Front Med (2022) 9:789623. doi: 10.3389/fmed.2022.789623 PMC9024201 35463014

[52] Zang S Chen Q Zhang Y Xu L Chen J . Comparison of the clinical effectiveness of AN69-oXiris versus AN69-ST filter in septic patients: A single-centre study. Blood Purification (2022) 51(7):617–29. doi: 10.1159/000519166 34610595

[53] Köhler T Schwier E Praxenthaler J Kirchner C Henzler D Eickmeyer C . Therapeutic modulation of the host defense by hemoadsorption with CytoSorb(®)-basics, indications and perspectives-a scoping review. Int J Mol Sci (2021) 22(23): 12786. doi: 10.3390/ijms222312786 34884590 PMC8657779

[54] Eichhorn T Rauscher S Hammer C Gröger M Fischer MB Weber V . Polystyrene-Divinylbenzene-Based adsorbents reduce endothelial activation and monocyte adhesion under septic conditions in a pore size-dependent manner. Inflammation (2016) 39(5):1737–46. doi: 10.1007/s10753-016-0408-1 PMC5023745 27503310

[55] McCrea K Ward R LaRosa SP . Removal of carbapenem-resistant enterobacteriaceae (CRE) from blood by heparin-functional hemoperfusion media. PloS One (2014) 9(12):e114242. doi: 10.1371/journal.pone.0114242 25469782 PMC4254995

[56] Kielstein JT Borchina DN Fühner T Hwang S Mattoon D Ball AJ . Hemofiltration with the seraph(®) 100 microbind(®) affinity filter decreases SARS-CoV-2 nucleocapsid protein in critically ill COVID-19 patients. Crit Care (London England) (2021) 25(1):190. doi: 10.1186/s13054-021-03597-3 PMC8169409 34074339

[57] Nishibori M Takahashi HK Katayama H Mori S Saito S Iwagaki H . Specific removal of monocytes from peripheral blood of septic patients by polymyxin b-immobilized filter column. Acta Med Okayama. (2009) 63(1):65–9. doi: 10.18926/AMO/31855 19247424

[58] Abe S Seo Y Hayashi H Matsuda K Usuki J Azuma A . Neutrophil adsorption by polymyxin b-immobilized fiber column for acute exacerbation in patients with interstitial pneumonia: A pilot study. Blood Purification (2010) 29(4):321–6. doi: 10.1159/000287232 20185904

[59] Ankawi G Fan W Pomarè Montin D Lorenzin A Neri M Caprara C . A new series of sorbent devices for multiple clinical purposes: Current evidence and future directions. Blood Purification (2019) 47(1-3):94–100. doi: 10.1159/000493523 30253409

[60] Lezhnina A Lem V Blatt N . Application of extracorporeal apheresis in treatment of COVID-19: A rapid review. BioNanoScience (2022) 12(3):979–93. doi: 10.1007/s12668-022-00987-x PMC9096332 35578681

[61] Dai X Zhang Y Yu L Jiang YA Chen L Chen Y . Effect of artificial liver blood purification treatment on the survival of critical ill COVID-19 patients. Artif Organs (2021) 45(7):762–9. doi: 10.1111/aor.13884 PMC8360150 33326621

[62] Xu K Cai H Shen Y Ni Q Chen Y Hu S . Management of COVID-19: the zhejiang experience. J Zhejiang Univ Med Sci (2020) 49(2):147–57. doi: 10.3785/j.issn.1008-9292.2020.02.02 PMC8800711 32391658

[63] Yessayan L Szamosfalvi B Napolitano L Singer B Kurabayashi K Song Y . Treatment of cytokine storm in COVID-19 patients with immunomodulatory therapy. ASAIO J (American Soc Artif Internal Organs 1992) (2020) 66(10):1079–83. doi: 10.1097/MAT.0000000000001239 PMC10660617 33136592

[64] Goldstein SL Askenazi DJ Basu RK Selewski DT Paden ML Krallman KA . Use of the selective cytopheretic device in critically ill children. Kidney Int Rep (2021) 6(3):775–84. doi: 10.1016/j.ekir.2020.12.010 PMC7938071 33732992

[65] Niazi NS Nassar TI Stewart IJ Honore PM Sharma K Chung KK . A review of extracorporeal blood purification techniques for the treatment of critically ill coronavirus disease 2019 patients. ASAIO J (American Soc Artif Internal Organs (1992) 68(10):1219–1227 doi: 10.1097/MAT.0000000000001761. PMC9521577 35417433

[66] Zang S Chen Q Zhang Y Xu L Chen J . Comparison of the clinical effectiveness of AN69-oXiris versus AN69-ST filter in septic patients: A single-centre study. Blood Purif (2021) 51(7):617–629. doi: 10.1159/000519166 34610595

[67] Padala SA Vakiti A White JJ Mulloy L Mohammed A . First reported use of highly adsorptive hemofilter in critically ill COVID-19 patients in the USA. J Clin Med Res (2020) 12(7):454–457. doi: 10.14740/jocmr4228 PMC7331860 32655741

[68] Zhang H Zhu G Yan L Lu Y Fang Q Shao F . The absorbing filter oxiris in severe coronavirus disease 2019 patients: A case series. Artif Organs (2020) 44(12):1296–302. doi: 10.1111/aor.13786 PMC7404740 32779737

[69] Premužić V Babel J Gardijan D Lapić I Gabelica R Ostojić Z . Extracorporeal blood purification is associated with improvement in biochemical and clinical variables in the critically-ill COVID-19 patients. Ther Apheresis Dialysis (2022) 26(2):316–29. doi: 10.1111/1744-9987.13730 PMC8652436 34486793

[70] Ugurov P Popevski D Gramosli T Neziri D Vuckova D Gjorgon M . Early initiation of extracorporeal blood purification using the AN69ST (oXiris(®)) hemofilter as a treatment modality for COVID-19 patients: A single-centre case series. Braz J Cardiovasc Surg (2022) 37(1):35–47. doi: 10.21470/1678-9741-2020-0403 33113325 PMC8973137

[71] Villa G Romagnoli S De Rosa S Greco M Resta M Pomarè Montin D . Blood purification therapy with a hemodiafilter featuring enhanced adsorptive properties for cytokine removal in patients presenting COVID-19: A pilot study. Crit Care (London England) (2020) 24(1):605. doi: 10.1186/s13054-020-03322-6 PMC7549343 33046113

[72] Kang K Luo Y Gao Y Zhang J Wang C Fei D . Continuous renal replacement therapy with oXiris filter may not be an effective resolution to alleviate cytokine release syndrome in non-AKI patients with severe and critical COVID-19. Front Pharmacol (2022) 13:817793. doi: 10.3389/fphar.2022.817793 35185571 PMC8854969

[73] Khadzhynov D von dem Berge U Muench F Karaivanov S Koerner R Kruse JM . Efficacy and complications of regional citrate anticoagulation during continuous renal replacement therapy in critically ill patients with COVID-19. J Crit Care (2022) 67:126–31. doi: 10.1016/j.jcrc.2021.10.010 PMC8576341 34768173

[74] Chiao C Faust H Singh T . Regional citrate and systemic heparin are adequate to maintain filter half-life for COVID-19 patients on continuous renal replacement therapy. Semin Dialysis (2022) 35(4):325‐9. doi: 10.1111/sdi.13061 PMC9115506 35141966

[75] Olynka Vega: A randomized, controlled trial to evaluate efficacy and safety of a highly selective semipermeable membrane (AN-69 oxiris) in comparison with a semi selective semipermeable membrane (Standard AN-69) in COVID-19 associated acute kidney injury: oXAKI-COV study. Available at: https://clinicaltrials.gov/ct2/show/NCT04597034 (Accessed June 18, 2022).

[76] Sekandarzad A Weber E Prager EP Graf E Bettinger D Wengenmayer T . Cytokine adsorption in patients with acute-on-chronic liver failure (CYTOHEP)-a single center, open-label, three-arm, randomized, controlled intervention trial. Trials (2022) 23(1):222. doi: 10.1186/s13063-022-06139-6 35303938 PMC8931566

[77] Gruda MC Ruggeberg KG O'Sullivan P Guliashvili T Scheirer AR Golobish TD . Broad adsorption of sepsis-related PAMP and DAMP molecules, mycotoxins, and cytokines from whole blood using CytoSorb® sorbent porous polymer beads. PloS One (2018) 13(1):e0191676. doi: 10.1371/journal.pone.0191676 29370247 PMC5784931

[78] Song T Hayanga J Durham L Garrison L McCarthy P Barksdale A . CytoSorb therapy in COVID-19 (CTC) patients requiring extracorporeal membrane oxygenation: A multicenter, retrospective registry. Front Med (Lausanne) (2021) 8:773461. doi: 10.3389/fmed.2021.773461 34988092 PMC8720923

[79] Stockmann H Thelen P Stroben F Pigorsch M Keller T Krannich A . CytoSorb rescue for COVID-19 patients with vasoplegic shock and multiple organ failure: A prospective, open-label, randomized controlled pilot study. Crit Care Med (2022) 50(6):964–76. doi: 10.1097/CCM.0000000000005493 PMC9112514 35135967

[80] Diab M Lehmann T Bothe W Akhyari P Platzer S Wendt D . Cytokine hemoadsorption during cardiac surgery versus standard surgical care for infective endocarditis (REMOVE): Results from a multicenter randomized controlled trial. Circulation (2022) 145(13):959–68. doi: 10.1161/CIRCULATIONAHA.121.056940 35213213

[81] Holmén A Corderfeldt A Lannemyr L Dellgren G Hansson EC . Whole blood adsorber during CPB and need for vasoactive treatment after valve surgery in acute endocarditis: A randomized controlled study. J Cardiothorac Vasc Anesth (2022) 36(8 Pt B):3015–0. doi: 10.1053/j.jvca.2022.02.028 35341666

[82] Rasch S Sancak S Erber J Wießner J Schulz D Huberle C . Influence of extracorporeal cytokine adsorption on hemodynamics in severe acute pancreatitis: Results of the matched cohort pancreatitis cytosorbents inflammatory cytokine removal (PACIFIC) study. Artif Organs (2022) 46(6):1019–6. doi: 10.1111/aor.14195 35182395

[83] Supady A Zahn T Kuhl M Maier S Benk C Kaier K . Cytokine adsorption in patients with post-cardiac arrest syndrome after extracorporeal cardiopulmonary resuscitation (CYTER) - a single-centre, open-label, randomised, controlled trial. Resuscitation (2022) 173:169–78. doi: 10.1016/j.resuscitation.2022.02.001 35143902

[84] Brouwer WP Duran S Kuijper M Ince C . Hemoadsorption with CytoSorb shows a decreased observed versus expected 28-day all-cause mortality in ICU patients with septic shock: A propensity-score-weighted retrospective study. Crit Care (2019) 23(1):317. doi: 10.1186/s13054-019-2588-1 31533846 PMC6749645

[85] Pieri M Fominskiy E Nardelli P Bonizzoni MA Scandroglio AM . CytoSorb purification in critically ill SARS-CoV-2 patients. Int J Artif Organs (2022) 45(2):216–20. doi: 10.1177/03913988211052572 34702109

[86] Ruiz-Rodríguez JC Chiscano-Camón L Ruiz-Sanmartin A Palmada C Paola Plata-Menchaca E Franco-Jarava C . Cytokine hemoadsorption as rescue therapy for critically ill patients with SARS-CoV-2 pneumonia with severe respiratory failure and hypercytokinemia. Front Med (Lausanne) (2021) 8:779038. doi: 10.3389/fmed.2021.779038 35083241 PMC8784514

[87] Rodeia SC Martins FL Fortuna P Bento L . Cytokine adsorption therapy during extracorporeal membrane oxygenation in adult patients with COVID-19. Blood Purif (2021) 51(9):791–8. doi: 10.1159/000518712 PMC8805080 34856539

[88] Nassiri AA Hakemi MS Miri MM Shahrami R Koomleh AA Sabaghian T . Blood purification with CytoSorb in critically ill COVID-19 patients: A case series of 26 patients. Artif Organs. (2021) 45(11):1338–47. doi: 10.1111/aor.14024 PMC8444787 34152629

[89] Wunderlich-Sperl F Kautzky S Pickem C Hörmann C . Adjuvant hemoadsorption therapy in patients with severe COVID-19 and related organ failure requiring CRRT or ECMO therapy: A case series. Int J Artif Organs (2021) 44(10):694–702. doi: 10.1177/03913988211030517 34256643

[90] Virág M Rottler M Ocskay K Leiner T Horváth B Blanco DA . Extracorporeal cytokine removal in critically ill COVID-19 patients: A case series. Front Med (Lausanne) (2021) 8:760435. doi: 10.3389/fmed.2021.760435 34869464 PMC8639689

[91] Jarczak D Roedl K Fischer M de Heer G Burdelski C Frings DP . Effect of hemadsorption therapy in critically ill patients with COVID-19 (CYTOCOV-19): A prospective randomized controlled pilot trial. Blood Purification (2022), 1–10. doi: 10.1159/000526446 PMC9747731 36075200

[92] Friesecke S Träger K Schittek GA Molnar Z Bach F Kogelmann K . International registry on the use of the CytoSorb® adsorber in ICU patients : Study protocol and preliminary results. Medizinische Klinik Intensivmedizin und Notfallmedizin (2019) 114(8):699–707. doi: 10.1007/s00063-017-0342-5 28871441

[93] Monard C Rimmelé T Ronco C . Extracorporeal blood purification therapies for sepsis. Blood Purification (2019) 47(Suppl 3):1–14. doi: 10.1159/000499520 30974444

[94] Bermejo-Martin JF González-Rivera M Almansa R Micheloud D Tedim AP Domínguez-Gil M . Viral RNA load in plasma is associated with critical illness and a dysregulated host response in COVID-19. Crit Care (London England) (2020) 24(1):691. doi: 10.1186/s13054-020-03398-0 PMC7734467 33317616

[95] Tang K Wu L Luo Y Gong B . Quantitative assessment of SARS-CoV-2 RNAemia and outcome in patients with coronavirus disease 2019. J Med Virol (2021) 93(5):3165–75. doi: 10.1002/jmv.26876 PMC8014647 33590923

[96] Kelly MM Wilkinson JD Rastegar M Lewis MS Betancourt J . Two patients with severe COVID pneumonia treated with the seraph-100 microbind affinity blood filter. J Intensive Care Med (2021) 36(10):1228–32. doi: 10.1177/08850666211039744 34516306

[97] Pape A Kielstein JT Krüger T Fühner T Brunkhorst R . Treatment of a critically ill COVID-19 patient with the seraph 100 microbind affinity filter. TH Open companion J to Thromb haemostasis (2021) 5(2):e134–e8. doi: 10.1055/s-0041-1727121 PMC8046512 33870077

[98] Rifkin BS Stewart IJ . Seraph-100 hemoperfusion in SARS-CoV-2-Infected patients early in critical illness: A case series. Blood Purification (2022) 51(4):317–20. doi: 10.1159/000517430 PMC8339049 34261058

[99] Sandoval D Rama I Quero M Hueso M Gómez F Cruzado JM . Treatment for severe COVID-19 with a biomimetic sorbent haemoperfusion device in patients on haemodialysis. Clin Kidney J (2021) 14(5):1475–7. doi: 10.1093/ckj/sfab010 PMC7929023 33953913

[100] Schmidt JJ Borchina DN Van't Klooster M Bulhan-Soki K Okioma R Herbst L . Interim analysis of the COSA (COVID-19 patients treated with the seraph® 100 microbind® affinity filter) registry. Nephrol dialysis Transplant (2022) 37(4):673–80. doi: 10.1093/ndt/gfab347 PMC8689741 34875087

[101] Shankaranarayanan D Muthukumar T Barbar T Bhasin A Gerardine S Lamba P . Anticoagulation strategies and filter life in COVID-19 patients receiving continuous renal replacement therapy: A single-center experience. Clin J Am Soc Nephrol CJASN (2020) 16(1):124–6. doi: 10.2215/CJN.08430520 PMC7792651 32943397

[102] Chitty SA Mobbs S Rifkin BS Stogner SW Lewis MS Betancourt J . A multicenter evaluation of the seraph 100 microbind affinity blood filter for the treatment of severe COVID-19. Crit Care Explorations (2022) 4(4):e0662. doi: 10.1097/CCE.0000000000000662 PMC9049035 35506015

[103] Carla Kikken-jussen: Safety and effectiveness evaluation of seraph 100 microbind affinity blood filter (Seraph 100) in the treatment of patients with COVID-19 . Available at: https://clinicaltrials.gov/ct2/show/NCT04547257 (Accessed June 18, 2022).

[104] Lu C Hou N . Skin hyperpigmentation in coronavirus disease 2019 patients: Is polymyxin b the culprit? Front Pharmacol (2020) 11:01304–. doi: 10.3389/fphar.2020.01304 PMC7494845 33013367

[105] Ronco C Reis T De Rosa S . Coronavirus epidemic and extracorporeal therapies in intensive care: si vis pacem para bellum. Blood Purification (2020) 49(3):255–8. doi: 10.1159/000507039 PMC7179535 32172242

[106] Srisawat N Tungsanga S Lumlertgul N Komaenthammasophon C Peerapornratana S Thamrongsat N . The effect of polymyxin b hemoperfusion on modulation of human leukocyte antigen DR in severe sepsis patients. Crit Care (London England) (2018) 22(1):279. doi: 10.1186/s13054-018-2077-y PMC6204024 30367647

[107] Johansson PI Windelov NA Rasmussen LS Sorensen AM Ostrowski SR . Blood levels of histone-complexed DNA fragments are associated with coagulopathy, inflammation and endothelial damage early after trauma. J Emergencies Trauma Shock (2013) 6(3):171–5. doi: 10.4103/0974-2700.115327 PMC3746437 23960372

[108] Peerapornratana S Sirivongrangson P Tungsanga S Tiankanon K Kulvichit W Putcharoen O . Endotoxin adsorbent therapy in severe COVID-19 pneumonia. Blood Purification (2022) 51(1):47–54. doi: 10.1159/000515628 33857940 PMC8089445

[109] Kuwana T Kinoshita K Hirabayashi M Ihara S Sawada N Mutoh T . PMX-DHP therapy for dyspnea and deoxygenation in severe COVID-19 pneumonia: A case series. Infection Drug Resistance (2021) 14:1305–10. doi: 10.2147/IDR.S299023 PMC8040694 33854342

[110] Shinomiya S Nakase K Fujii A Takahara Y Adachi H Okuro M . Tocilizumab and PMX-DHP have efficacy for severe COVID-19 pneumonia. SAGE Open Med Case Rep (2021) 9:2050313x21991063. doi: 10.1177/2050313X21991063 PMC7970680 33796310

[111] Kusaba Y Izumi S Takasaki J Suzuki M Katagiri D Katsuno T . Successful recovery from COVID-19-associated acute respiratory failure with polymyxin b-immobilized fiber column-direct hemoperfusion. Internal Med (Tokyo Japan) (2020) 59(19):2405–8. doi: 10.2169/internalmedicine.5413-20 PMC7644505 32863364

[112] Ishiwari M Togashi Y Takoi H Kikuchi R Kono Y Abe S . Polymyxin b haemoperfusion treatment for respiratory failure and hyperferritinaemia due to COVID-19. Respirol Case Rep (2020) 8(9):e00679. doi: 10.1002/rcr2.679 33163186 PMC7604553

[113] Katagiri D Ishikane M Asai Y Izumi S Takasaki J Katsuoka H . Direct hemoperfusion using a polymyxin b-immobilized polystyrene column for COVID-19. J Clin apheresis (2021) 36(3):313–21. doi: 10.1002/jca.21861 PMC8246724 33325084

[114] De Rosa S Cutuli SL Ferrer R Antonelli M Ronco C . Polymyxin b hemoperfusion in coronavirus disease 2019 patients with endotoxic shock: Case series from EUPHAS2 registry. Artif Organs (2021) 45(6):E187–e94. doi: 10.1111/aor.13900 33377184

[115] Pomarè Montin D Ankawi G Lorenzin A Neri M Caprara C Ronco C . Biocompatibility and cytotoxic evaluation of new sorbent cartridges for blood hemoperfusion. Blood Purification (2018) 46(3):187–95. doi: 10.1159/000489921 29886501

[116] Lezhnina A Lem V Blatt N . Application of extracorporeal apheresis in treatment of COVID-19: A rapid review. BioNanoScience (2022) 12(3):979–93. doi: 10.1007/s12668-022-00987-x PMC9096332 35578681

[117] Surasit K Srisawat N . The efficacy of early additional hemoperfusion therapy for severe COVID-19 patients: A prospective cohort study. Blood Purification (2022) 51(11):879–888. doi: 10.1159/000521713 PMC9059038 35139519

[118] Alavi Darazam I Kazempour M Pourhoseingholi MA Hatami F Rabiei MM Javandoust Gharehbagh F . Efficacy of hemoperfusion in severe and critical cases of COVID-19. Blood Purif (2022), 1–9. doi: 10.1159/000524606 PMC9393767 35580567

[119] Abdullayev R Gul F Bilgili B Seven S Cinel I . Cytokine adsorption in critically ill COVID-19 patients, a case-control study. J Intensive Care Med (2022) 37(9):1223–8. doi: 10.1177/08850666221085185 PMC8919098 35274999

[120] Ronco C Bagshaw SM Bellomo R Clark WR Husain-Syed F Kellum JA . Extracorporeal blood purification and organ support in the critically ill patient during COVID-19 pandemic: Expert review and recommendation. Blood Purif (2021) 50(1):17–27. doi: 10.1159/000508125 32454500 PMC7270067

[121] Shokouhi S Barati S Kazeminia N Jamali F Roshan B Sahraei Z . Evaluating the elimination status of medications used for COVID-19 during hemoperfusion and therapeutic plasma exchange: A review. Int immunopharmacol (2021) 97:107707. doi: 10.1016/j.intimp.2021.107707 33962224 PMC8059942

[122] Chen L Chen Y Chen Y Cheng M Ding X Dou X . Translation: Expert consensus on the application of artificial liver blood purification system in the treatment of severe and critical COVID-19. Infect Microbes Dis (2020) 36(6):1228–9. doi: 10.3969/j.issn.1001- PMC7227200 40479212

[123] Xia Q Xu K Yu L Zhang H Li L . Application value of artificial liver support system in the treatment of cytokine storm in patients with COVID-19. Int Immunopharmacol (2021) 90:107120. doi: 10.1016/j.intimp.2020.107120 33162345 PMC7604162

[124] Pino CJ Yevzlin AS Tumlin J Humes HD . Cell-based strategies for the treatment of kidney dysfunction: A review. Blood Purification (2012) 34(2):117–23. doi: 10.1159/000341649 PMC3836365 23095410

[125] Selig DJ Reed T Chung KK Kress AT Stewart IJ DeLuca JP . Hemoperfusion with seraph 100 microbind affinity blood filter unlikely to require increased antibiotic dosing: A simulations study using a Pharmacokinetic/Pharmacodynamic approach. Blood Purif (2022) 1–7. doi: 10.1159/000524457 35526522

